# Validation of the RUDAS for the Identification of Dementia in Illiterate and Low-Educated Older Adults in Lima, Peru

**DOI:** 10.3389/fneur.2020.00374

**Published:** 2020-05-05

**Authors:** Nilton Custodio, Rosa Montesinos, David Lira, Eder Herrera-Perez, Kristhy Chavez, Willyams Reynoso-Guzman, Maritza Pintado-Caipa, José Cuenca, Carlos Gamboa, Tatiana Metcalf

**Affiliations:** ^1^Servicio de Neurología, Instituto Peruano de Neurociencias, Lima, Peru; ^2^Unidad de diagnóstico de deterioro cognitivo y prevención de demencia, Instituto Peruano de Neurociencias, Lima, Peru; ^3^Unidad de Investigación, Instituto Peruano de Neurociencias, Lima, Peru; ^4^Servicio de Rehabilitación, Instituto Peruano de Neurociencias, Lima, Peru; ^5^Grupo de investigación Molident, Universidad San Ignacio de Loyola, Lima, Peru; ^6^Atlantic Fellow, Global Brain Health Institute, University of California, San Francisco, San Francisco, CA, United States; ^7^Servicio de Neuropsicología, Instituto Peruano de Neurociencias, Lima, Peru; ^8^Carrera de Psicología, Facultad de Ciencias de la Salud, Universidad Privada del Norte, Lima, Peru

**Keywords:** mild cognitive impairment, neuropsychology, dementia, neurocognitive disorders, Alzheimer's disease, brief cognitive assessment, illiteracy

## Abstract

**Objectives:** To evaluate the performance of the Peruvian version of the Rowland Universal Dementia Assessment Scale (RUDAS-PE) in discriminating between controls and patients with mild cognitive impairment (MCI) and dementia in an illiterate population with low-levels of education.

**Methods:** We compared the cognitive performance of 187 elderly subjects who were illiterate (controls *n* = 60; MCI *n* = 64; dementia *n* = 63). Neuropsychological measures included the RUDAS-PE, Mini-Mental State Examination (MMSE), INECO Frontal Screening (IFS), and Pfeffer Functional Activities Questionnaire (PFAQ). The results were compared to a neuropsychological evaluation (gold standard), including use of Clinical Dementia Rating (CDR) scores.

**Results:** We found a Cronbach's alpha was 0.65; Spearman's correlation coefficient was 0.79 (*p* < 0.01). The area under the receiver operating characteristics curve for the RUDAS to discriminate dementia from MCI was 98.0% with an optimal cut-off <19 (sensitivity 95%, specificity 97%); whereas, to differentiate MCI and controls was 98.0% with an optimal cut-off <23 (sensitivity 89%, specificity 93%).

**Conclusions:** Based on its excellent psychometric properties, we find the RUDAS-PE suitable to aid in the opportune detection of dementia in a geriatric illiterate population with low-levels of education.

## Introduction

Illiteracy rates among youth (age 15 to 24 years) and adults are decreasing worldwide. In Peru, data from the Instituto Nacional de Estadística e Informática (INEI) show that illiteracy rates among persons age 15 years and older remain high: 5.9% (1). Moreover, illiteracy among adults age 60 years and older are highest in rural areas (41.6% rural vs. 12.3% urban) and higher among females (27.5 vs. 8.2% in males) (2). Studies show that 21.9% of the population in Peru has an elementary level of education; the majority of which is found among rural inhabitants (43 vs. 16% urban) (2).

It is common to find large portions of the older population experience limited access to health care systems, whether as a result of age discrimination or other barriers such as cost. This raises a high concern as the health and medical needs for this age group, particularly in the ability to detect cognitive deterioration and dementia (3, 4), are largely unmet (5). In Peru, this is primarily due to a lack of validated and standardized instruments to evaluate cognition and functionality in marginalized populations, i.e., low-levels of education and literacy rates, rural communities, indigenous groups or populations where multiple languages exist in addition to Spanish (6, 7).

Many attempts have been made (4) with the purpose of detecting dementia in illiterate populations in low-educational settings. The Cognitive State Test (COST) seems to give acceptable results (3). Unfortunately, like the Mini-Mental State Examination (MMSE) (8–10), the COST fails to address mild cognitive impairment (4). In Peru, the Memory Alteration Test (M^@^T) results have only been reported in individuals of low-educational backgrounds (at least 4 years of regular education) (11) and can distinguish patients with amnestic mild cognitive impairment (aMCI) from controls and patients with early stage Alzheimer's disease [AD]. Yet, since it can only evaluate memory and orientation, the M^@^T is unable to detect other types of dementia.

Within the framework of this criteria, we look to validate the Rowland Universal Dementia Assessment Scale (RUDAS). The RUDAS is a BCT that has proven itself a useful instrument for the detection of dementia in an illiterate population within a primary care setting (12) as well as in populations with a low-level of education (13, 14). It is a simple instrument, consisting of six-items that explore recent verbal memory, visuo-spatial orientation, motor praxis, visuo-constructive praxis, judgment, and language. Like the MMSE, it has an optimal score of 30 points, where lower scores suggest severe cognitive impairment (12). It has been proposed that the RUDAS has reasonable psychometric characteristics and is particularly useful in patients of various languages and cultures, thereby being preferable in populations with low-levels of educational attainment. While it is true that the RUDAS is validated in an urban Peruvian population with a middle-level of education (15), it has yet to be adapted for and evaluated in an illiterate elderly population with low-levels of education.

## Methods

### Study Design

This is a diagnostic accuracy study designed focusing on an urban illiterate population with the following objectives:

1. Establish the sensitivity and specificity of the RUDAS.
2. Establish the parameters for the RUDAS to discriminate MCI and dementia.
3. Compare the capacity of the RUDAS and MMSE to discriminate between normal cognitive function and patients with MCI and dementia.

### Participants

This study took place in regional health clinics within Lima, Peru. A previous awareness campaign on risk factors involved in cognitive impairment served as our primary source of patient recruitment within the Ventanilla community. Potential research participants included those who regularly assisted the scheduled activities designed to evaluate cognitive impairment as part of the pre-screening process. After having passed a screening test and neuropsychological evaluation, individuals were then allocated into three groups for further statistical analysis:

1. Control group: Individuals without cognitive impairment (no-CI) (CDR = 0).
2. Mild Cognitive Impairment (MCI) group: Individuals with clinical and neuropsychological criteria of MCI (CDR = 0.5).
3. Dementia group: Individuals with clinical and neuropsychological criteria compatible with dementia in its initial stages (CDR = 1 and 2).

### Inclusion Criteria

1. Participants were selected according to the following criteria:
2. Males and females aged 60 and above.
3. Illiterate persons of at least 15 years old defined as one with no education (< 1 full year of formal education completed, and inability to read or write). Also, individuals without prior literacy experience who participate in basic adult education programs or “night school” on a regular basis after having turned 60).
4. Individuals who are native Spanish speakers or persons who speak Spanish as a second language > 10 years.
5. Individuals diagnosed with aMCI based on the Diagnostic and Statistical Manual of Mental Disorders, fifth edition (DSM-5) and Clinical Dementia Rating (CDR) criteria.
6. Individuals diagnosed with signs of dementia according to DSM-5 and CDR criteria.

### Exclusion Criteria

Participants were excluded if they had difficulty performing the cognitive tests due to auditory or visual problems, or any other physical problems that interfered with their performance; if they were considered functionally literate (defined as those who received a non-formal education for a minimum of 4 years before the age of 15, are able to read, write, do mathematical calculations, and are socially functional); or did not speak or understand Spanish. We further excluded patients who were diagnosed/or had symptoms compatible with advanced stage dementia or another psychiatric illness (bipolar disorder, psychosis, schizophrenia, and personality disorders) as well as participants diagnosed with concomitant cerebrovascular pathologies, mental retardation, traumatic brain injury sequelae, depression (according to the Beck Depression Inventory-II), had a history of addiction or substance abuse, or who in the last seven consecutive days prior to the evaluation had taken any of the following medications: opioids, decongestants, anti-spasmodics, anti-cholinergics, anti-arrhythmic, anti-depressants, anti-psychotics, such as valproate, phenobarbital, fentanyl, carbamazepine, and levetiracetam. In cases where patients would take these medications for a chronic illness, and only if their medical condition would allow it, it was recommended to stop their medication for seven consecutive days prior to commencing the brief cognitive assessment.

### Ethical Aspects

This study was conducted in accordance with the Council for International Organizations and Medical Sciences guidelines. All participants signed a consent form prior to the study in accordance with the Declaration of Helsinski. The protocol was approved by the ethics committee at the Instituto de Medicina Tropical “Daniel Alcides Carrión” of the “Universidad Nacional Mayor de San Marcos” approval number CIEI-2018-020.

### Measures

#### Rowland Universal Dementia Assessment Scale (RUDAS)

##### Validation of the RUDAS in an urban population with a mid level education

The RUDAS has recently been evaluated in patients age ≥60 years with a mid-level education in Peru. The optimal cut-off for ED and MCI was <21 with a sensitivity of 90.2% and specificity of 73.8%; and <24 between MCI and controls with a sensitivity of 96% and specificity of 90.2% (15).

##### RUDAS-PE adaptation for illiterate population

The adaptation process corresponded to the standards and methodology of the RUDAS-PE set by Custodio et al. (15) and based on the Spanish translated version of the RUDAS by Ramos-Ríos et al. (14). Suggestions made by clinical experts (neurologists SC and DL, and neuropsychologists JC and MS) were used to make improvements to the existing RUDAS-PE. Only minor procedural changes to the administration of the test were made: (1) state the precise time allotted for the administrator to demonstrate and evaluate the alternating hands portion of the motor praxis section, and (2) instruct the test administrator on the specific size of the cube in the cube-drawing portion of the visuo-constructional section of the test as follows, “take a sheet of A4 paper and draw a cube with lateral edges of 12 cm in length at a 45° angle.” These recommendations were approved and introduced into the final version of the RUDAS-PE (Supplemental Material 1).

##### Pilot study (RUDAS in a healthy illiterate elderly population)

Convenience sampling was used to select a group of 30 cognitively healthy illiterate adults (average age 69; no more than 01 year of schooling) from a local senior center. Literacy was self-reported (“Are you able to read or write?”). Cognitive health was based on a standardized neuropsychological evaluation. This study allowed us to verify the validity of the content and criteria.

#### Mini-Mental State Examination (MMSE)

The MMSE is a BCT that that briefly evaluates cognitive function via 5 main sections: orientation, registration, attention and calculation, recall, and language. A Peruvian version was adapted and validated (modified from the Argentinian version) (16) incorporating cultural modifications specific to Peru. Subsequent studies in Peruvian seniors where most participants were illiterate showed a low sensitivity of 64.1%, a specificity of 84.1%, and a high proportion of false positives (15.9%) (17) indicating that the MMSE is not a good screening test for any type of dementias in geriatric populations.

#### INECO Frontal Screening Test

The IFS test evaluates executive functions taking ~10 min to conduct. Its maximum score is 30 points (8 subtests): motor programming (3 points), motor inhibitory control (3 points), backward digital span (6 points), verbal working memory (2 points), spatial working memory (4 points), abstraction capacity (3 points), and verbal inhibitory control (6 points). A Peruvian version of the IFS showed a sensitivity of 94.12% and specificity of 94.2% (18).

### Gold Standard

The neuropsychological evaluation that confirmed the cognitive state of the study groups (no-CI, MCI, and dementia) consisted of a battery of tests adapted for use in the Peruvian population. The decision criteria to determine cognitive impairment were two standard deviations less than the average. The neuropsychological battery included the following: DSM-5 criteria, the PFAQ, and CDR.

#### Diagnostic and Statistical Manual of Mental Disorders (DSM-5)

In the current edition (5th) of the DSM, the diagnostic criteria for neurocognitive disorders (NCD) moves away from the current concept of dementia and MCI taking into account all causes of cognitive impairment irrespective of age group. It is comprised of delirium and two syndromes: major NCD (representing dementia) and minor NCD (representing MCI stage), depending on functionality.

#### Pfeffer Functional Activities Questionnaire (PFAQ)

The PFAQ is a test that includes 11 questions about daily activities involving money management, shopping, heating water, preparing a meal, staying up-to-date on current events, discussing TV/radio/newspapers, remembering appointments and medication, and traveling outside the neighborhood. Scoring rages from 0 to 3 according to severity of disability in each activity. The maximum score is 33, where a cutoff of seven indicates impaired function (19).

#### Clinical Dementia Rating (CDR) Scale

The CDR is a global assessment tool (often referred to as global CDR) that was first introduced in the early 1980's to evaluate mild senile dementia of AD (20) and is currently used to measure social changes, behaviors, and functions of the patient. The score is designed to stage dementia severity and is based on independently semi-structured interviews of patients and informants as well as clinical judgment from the treating physician. It is calculated based on 6 cognitive and behavioral domains including memory, orientation, judgment and problem solving, community affairs, home and hobbies performance, and personal care. It has many advantages: it is independent of other psychometric tests, it does not need a baseline evaluation, it can be used as a control for each individual. Moreover, it has good inter-rater reliability, concurrent validity, predictive validity, and clinical-neuropathological correlation in AD. Its disadvantages include special training requirements, the right skills and good judgment of the interviewer to obtain the pertinent information, and the length of time it takes to be administered (at least 30 min).

### Methodological Definition of Illiteracy/Illiterates

A person ≥ 60 years was identified as illiterate by:

1. Determining the years of education attained by asking the patient, “How many years did you go to school?”
2. Asking those with <1 year of formal schooling “Are you able to read or write?”
3. Having those who stated that they could write or read a few words (name and place of birth), confirm they could read a simple phrase.

Participants were selected if they had 0 to < 1 year of schooling and did not know how to read nor write.

### Medical Protocol

Random sampling was used to select participants. Subject assent to participate was registered using participant's digital fingerprint and a signature of informed consent from the caregiver/informant, having already been reviewed and approved by the proper regulatory authorities.

### Clinical Evaluation

A trained interviewer conducted the clinical evaluation. These procedures included the following: (1) demographic information (via interview and standardized neurologic examination found in the case report forms, (2) anthropometric measures and blood pressure, and (3) comorbidity data and medical treatment received 1 week prior to evaluation.

### BCTs and Parametric Test Measurements

Cognitive decline was evaluated in three successive phases: (1) screening - to detect cases with cognitive decline; (2) nosological diagnosis - to determine the specific cause of cognitive decline; (3) final classification of the subjects into their respective group according to their clinical state: controls, MCI, and dementia.

#### Screening Phase

Field evaluators conducted a clinical neurologic assessment that included anthropometric measurements and blood pressure. Medications taken a week before were recorded as well as responses from their respective caregivers/informants to a subjective memory complaint questionnaire (SMCQ) (questionnaire of memory deficits of everyday life). The PFAQ and BCTs (MMSE, IFS and RUDAS-PE) were administered for the first time.

The cut-off points for this study protocol were as follows: MMSE < 22 for those with 1 to 3 years of education and MMSE < 18 for illiterates; PFAQ > 7.

#### Nosological Diagnosis: Parametric Tests

All study participants were evaluated twice with <5-week interval between assessments. This time interval (mean 37 ± days) was defined to yield a higher reliability coefficient. Whenever a BCT was positive for cognitive decline during the screening phase, it was repeated by a different evaluator (neurologist or geriatrician) in the diagnostic phase. Confirmed cases were then identified as patient with cognitive impairment (PCI). In this phase, additional parametric tests were administered to rule-out cognitive impairment from neurodegenerative causes including the Hachinski modified ischemic scale questionnaire, the BDI-II and subsequent RUDAS-PE, MMSE, IFS, and PFAQ tests.

#### Final Classification: Parametric Tests

The CDR scale was applied by a panel of two evaluators specializing in neuro-rehabilitation and neuropsychology each of whom were blinded to each other's clinical assessments. Next we applied the DSM-5 criteria for major and minor NCDs (corresponding to our study definitions of dementia and MCI, respectively) and the CDR assessment to help determine which stage of dementia the participants were experiencing. The CDR analysis was based on a scale of 0–2: controls (CDR = 0); MCI (CDR = 0.5), early stage dementia (CDR = 1), and moderate stage dementia (CDR = 2). CDR score was applied to both participants as well as to their caregivers/informants. In cases where the assignment of CDR for dementia staging was questionable, a panel consisting of neurologists, geriatricians, neuro-rehabilitators, and neuropsychologists would reach a consensus. Participants who did not present subjective memory complaints on the SMCQ about daily life and also had normal results on all BCTs were considered cognitively healthy and became part of the control group. Evaluators were blinded to a structured neuropsychological evaluation in the third phase. RUDAS-PE results did not form part of the neuropsychological battery used to diagnose and classify subjects into their respective study groups: control, MCI, and dementia.

The evaluation team of the second and third phase (neurologists and neuropsychologist with advanced training in dementia research) were different from the team in the first phase (geriatric residents, psychology and neuroscience students under supervision by a neurologist and medical rehabilitators – also experts in dementia). Throughout the study, experts that applied the neuropsychological tests (gold standard) were blind to the BCTs results.

### Data Analysis

Stata version 2.0 (StataCorp LLC, College Station, Texas) was used for data analysis. A descriptive statistical analysis was performed on the demographic and clinical characteristics of the study population as well as on the psychometric properties of the BCTs. *P* < 0.05 were considered statistically significant.

### Demographic Characteristics

We applied two-tailed *t*-tests (discrete variables) and Chi Square test (categorical variables) for between-group comparisons.

### Psychometric Properties

#### Reliability

Reliability was tested during the diagnostic phase. Cronbach's alpha coefficient was used to calculate homogeneity and internal consistency. We removed subsequent domains of the RUDAS-PE to evaluate the changes in the coefficient. Lin's concordance correlation coefficient (CCC) and Bland-Altman plots were used to assess test-retest reliability of the RUDAS scores administered to the same population during the first and second phase; the time interval between the two evaluations was <5 weeks. Lastly, inter-rater reliability was also calculated.

#### Construct Validity

An expert panel of judges consisting of four dementia experts (neurologist SC and DL; neuropsychologists JC and MS) experienced in conducting cognitive and neuropsychological assessments examined the content validity of constructs. A content validity questionnaire (Supplemental Material 2) assessed construct-item match and language group suitability.

#### Criterion-Related Validity: Concurrent, Convergent, and Discriminant

During the second phase, given the non-normally distributed data, concurrent validity was assessed by determining Spearman's rank correlation coefficient (ρ) to measure the strength of a monotonic relationship between paired data: RUDAS-PE/MMSE, RUDAS-PE/IFS, RUDAS-PE/PFAQ, and the RUDAS-PE/CDR, namely for the total RUDAS scores and its cognitive domains in each of these paired test comparisons.

The following Spearman's correlation classification was used:

- 0.0–0.25 “very weak”
- 0.26–50 “weak”
- 0.51–0.75 “moderate to strong”
- 0.76–1.0 “very strong to perfect”.

We used logistic regression (logit) for each of the three study group pairs (dementia in early stages/MCI, MCI/control, and dementia in early stages/control) using a two-variable model: final diagnosis as dependent variable, and each BCT as independent variable. For discriminant validity we measured the average of the sum score of the RUDAS-PE and the average score for each of its domains in each of the three groups (controls, MCI, and dementia). These were then compared using the Independent Samples *t*-test. We also analyzed the percentage of individuals correctly classified and conducted a multivariate analysis of variance (MANOVA).

#### Diagnostic Accuracy

Diagnostic accuracy was evaluated via a post-estimation analysis to configure the Receiver Operating Characteristic (ROC) curves including calculation of the area under the ROC curve (AUC). The maximum values were used to establish sensitivity, specificity, and predictive values. Finally, we compared the AUCs of these BCTs using the Handley and McNeil method.

## Results

### Participants

**Figure 1:**
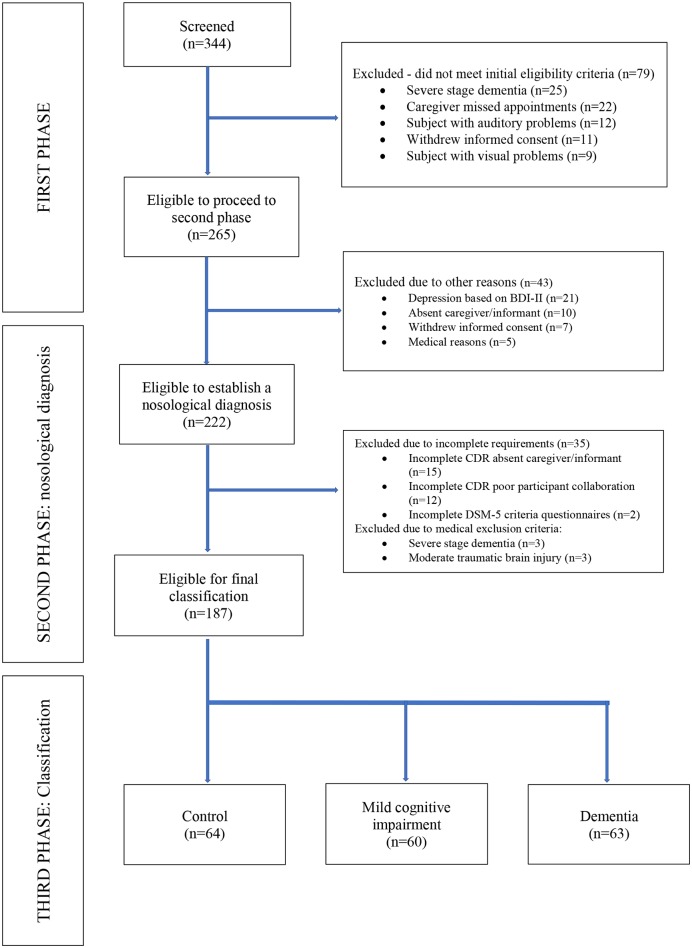
Study flow diagram. Centros de Salud de Ventanilla, Callao, Lima. 2018-2019.

The study began with 344 participants; 79 of whom were lost to follow-up: BCTs identified participants with severe stages of dementia (*n* = 25), difficulty attending scheduled visits (*n* = 22), hearing problems (*n* = 12), withdrew informed consent (*n* = 11), and visual problems (*n* = 9). In the second phase, 43 of the 265 participants were excluded: met BDI-II criteria for depression (*n* = 21), absent caregiver/informant (*n* = 10), withdrew informed consent (*n* = 7), and medical reasons (*n* = 5). Thus, 222 participants completed the second phase. An additional 35 participants were excluded in the current analysis for the following reasons: incomplete CDR interview due to absent caregiver (*n* = 15), incomplete CDR interview due to poor collaboration among study participants (*n* = 12), incomplete DSM-5 diagnostic criteria questionnaires (*n* = 2), severe stage dementia (*n* = 3), and moderate traumatic brain injury (*n* = 3) (Figure 1).

### Clinical and Demographic Profiles

A little over half (56.15%) of the study sample were women. The proportion of females was similar for each study group: dementia (55.6%), MCI (56.7%), and (56.2%) control; there was no significant difference found between study groups. The average age was 70.14 ± 3.79; the control group was significantly younger than the dementia group (*p* = 0.000). Likewise, the MCI group was significantly younger than the dementia group (*p* = 0.000); there were no significant differences in age between the control and MCI groups (*p* = 0.794). All three BCTs (MMSE, IFS, and RUDAS-PE) showed less performance in the dementia group as compared to MCI and control groups. Similarly, MCI patients performed less than the controls. The RUDAS-PE score for the dementia group was 14.97 ± 2.21, 20.43 ± 1.39 for MC,I and 23.87 ± 0.93 for controls. The BDI-II score for the sample population was 6.46 ± 2.98. The BDI-II score in the dementia group was 7.24 ± 3.06, 6.20 ± 2.94 for MCI, and 5.94 ± 2.82 for controls (Table 1). None of the groups met the criteria for depression; there was no significant difference between each of the groups based on BDI-II score.

**Table 1:** Demographic characteristics and brief cognitive test performance according to study groups.

	**Control (*n* = 64)**	**MCI (*n* = 60)**	**Dementia (*n* = 63)**	***p*-value 1 (control vs. MCI)**	***p*-value 2 (MCI vs. Dem)**	***p*-value 3 (control vs. Dem)**
Female (%)	36 (56.2)	34 (56.7)	35 (55.6)	0.554	0.523	0.540
Age in years, mean (SD)	68.92 (3.45)	68.77 (3.14)	72.69 (3.42)	0.794	0.000^**^	0.000^**^
MMSE score, mean (SD)	20.16 (1.49)	17.85 (1.64)	10.11 (1.58)	0.000^**^	0.000^**^	0.000^**^
IFS score, mean (SD)	24.06 (1.11)	19.9 (1.34)	14.25 (1.96)	0.000^**^	0.000^**^	0.000^**^
RUDAS-PE score, mean (SD)	23.87 (0.93)	20.43 (1,39)	14.97 (2.21)	0.000^**^	0.000^**^	0.000^**^
BDI-II Score, mean (SD)	5.94 (2.82)	6.20 (2.94)	7.24 (3.06)	0.613	0.058	0.014^*^

*Centros de Salud de Ventanilla, Callao, Lima. 2018-2019*.

*BCTs, brief cognitive tests; MCI, Mild Cognitive Impairment; Dem, Dementia; SD, standard deviation; MMSE, Mini Mental State Examination; IFS, INECO Frontal Screening; RUDAS-PE, Rowland Universal Dementia Assessment Scale, Peruvian version; BDI-II, Beck Depression Inventory-second edition*.

* p < 0.05;

** *p < 0.001*.

### Psychometric Properties of the RUDAS-PE

#### Internal Consistency

Internal consistency was calculated among all 187 participants completing the third phase. Cronbach's alpha for the RUDAS-PE in a geriatric illiterate population was 0.65. When a RUDAS-PE dominion was removed, the Cronbach alpha coefficient did not increase, on the con trary, the value decreased. For this reason, all the dominions showed to positively contribute to the RUDAS-PE and were consistent throughout the test.

#### Test-Retest Reliability

Lin's concordance correlation coefficient was used to evaluate the test-retest reliability of the RUDAS-PE based on the scores obtained during the first and second phase of the study (Table 2). The total average scores of the RUDAS-PE in the two times that the test was administered were similar (20.08 vs. 19.76); the differences between the two were close to zero (0.32). Meanwhile, Bland-Altman plots showed that the mean differences of the test and re-test included zero for the RUDAS-PE, indicating no significant difference between the two measurements. On the other hand, CCC showed a moderate positive correlation (0.61) between the two observations. Similar patterns were recorded for each dominion of the RUDAS-PE, indicating overall acceptable test-retest reliability.

**Table 2:** Test-Retest Reliability of the Rowland Universal Dementia Assessment Scale (RUDAS-PE).

**Cognitive domain**	**Phase 1(test)**		**Phase 2(retest)**		**ρ_c_ (95% CI)**	**Difference**	**Bland and altmanlimits (95% CI)**
	**Avg**	**SD**	**Avg**	**SD**			
RUDAS-PE total score	20.08	3.42	19.76	3.78	0.61 (0.52–67)	−0.32	−8.59–9.21
Memory	5.24	2.27	5.08	2.11	0.21 (0.12–0.30)	−0.16	−2.84–2.54
Visuo-spatial orientation	4.79	0.62	4.56	0.50	0.54 (0.46–0.61)	0.23	-3.21–3.69
Motor praxis	1.85	0.59	1.66	0.48	0.28 (0.18–0.37)	−0.19	−2.15–1.76
Visuo-spatial construction	0.53	0.56	0.69	0.64	0.25 (0.15–0.33)	0.16	−2.12–1.05
Judgment	1.75	0.67	1.60	0.88	0.44 (0.36–0.51)	−0.15	−2.27–2.06
Language	6.54	1.13	6.92	0.98	0,24 (0.15–0.36)	0.38	−2.92–3.12

*Centros de Salud de Ventanilla, Callao, Lima. 2018-2019*.

*RUDAS-PE, Rowland Universal Dementia Assessment Scale, Peruvian version; SD, standard deviation; Avg, average; CI, confidence interval; ρ_c_, Lin's concordance correlation coefficient*.

Intraclass correlation coefficients (ICCs) were also used to assess test-retest reliability resulting in an ICC of 0.96. The correlation between first and second evaluation was 95.9%. The correlation for the RUDAS-PE total score for every dominion were above 40%.

#### Criterion-Related Validity: Concurrent, Convergent, and Discriminant

Strong correlations were found between the RUDAS-PE/MMSE (ρ = 0.86; SD: 0.14, CI 95%), RUDAS-PE/IFS (ρ = 0.87; SD: 0.09, CI 95%), RUDAS-PE/PFAQ (ρ = 0.83; SD: 0.27, CI 95%), and RUDAS-PE/CDR (ρ = 0.86; SD: 0.18, CI 95%).

#### Discriminant Validity

**Figure 2:**
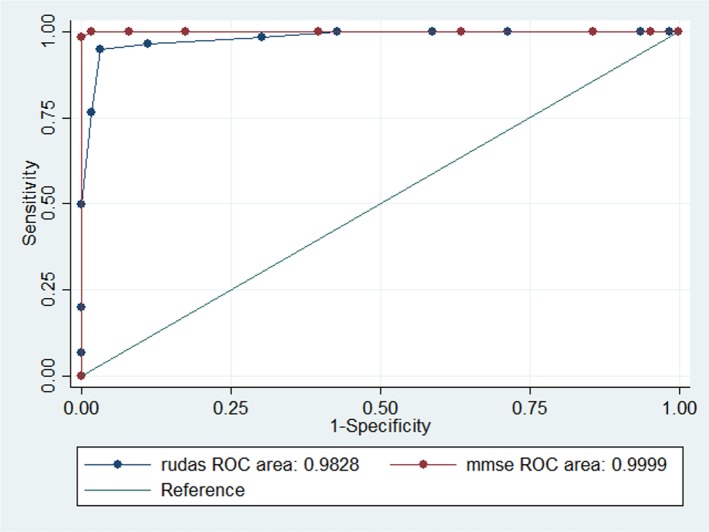
Receiver-operating characteristic (ROC) curve for the Peruvian version of the Rowland Universal Dementia Assessment Scale (RUDAS-PE) and the Mini Mental State Examination (MMSE) in 124 patients for discrimination between mild cognitive impairment (MCI) and control groups. Centros de Salud de Ventanilla, Callao, Lima. 2018-2019.

**Figure 3:**
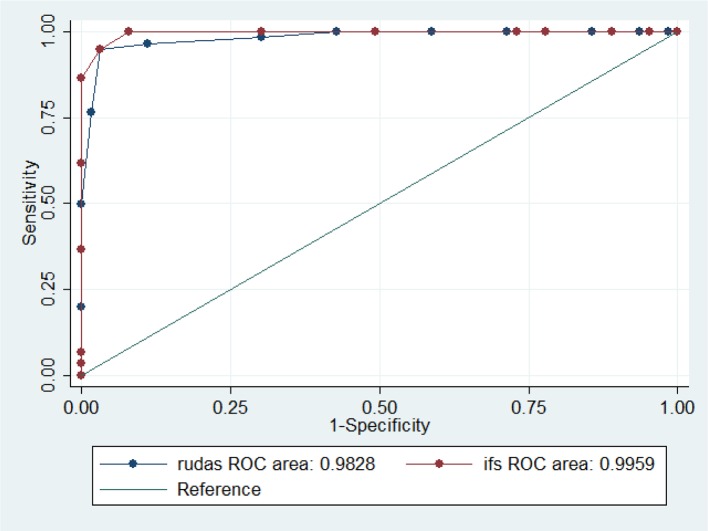
Receiver-operating characteristic (ROC) curve of the Peruvian version of the Rowland Universal Dementia Assessment Scale (RUDAS-PE) and the INECO Frontal Screening (IFS) in 124 patients for discrimination between mild cognitive impairment (MCI) and control groups. Centros de Salud de Ventanilla, Callao, Lima. 2018-2019.

**Figure 4:**
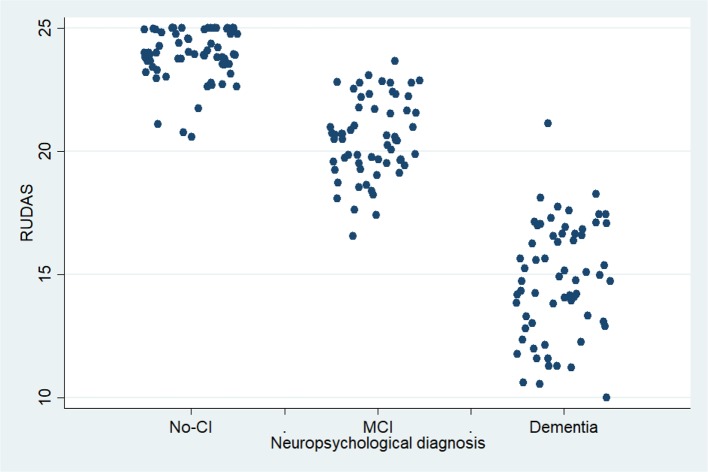
Scores distribution according to neuropsychological diagnosis for the Peruvian version of the Rowland Universal Dementia Assessment Scale (RUDAS-PE), (*n* = 187). Centros de Salud de Ventanilla, Callao, Lima. 2018-2019.

There was no overlap in the RUDAS-PE scores as depicted in the dispersion graph (Figure 2), indicating a very good ability to discriminate between dementia, MCI, and healthy controls. For each BCT, an AUC-ROC curve was calculated for each of the following study groups: (1) control vs. MCI (*n* = 124), (2) control vs. dementia (*n* = 127), and (3) MCI vs. dementia (*n* = 123). The comparison results in the control group and the dementia group showed each of the tests (RUDAS-PE, IFS, and MMSE) approaching an AUC of 1. Similarly, comparing the RUDAS-PE, IFS, and MMSE between the controls and MCI showed an AUC of 1. Figure 2 shows the ROC curve for the RUDAS-PE (AUC = 0.9828) compared against MMSE (AUC = 0.9999) to discriminate between MCI and controls; meanwhile, Figure 3 shows the ROC curve of the RUDAS-PE (AUC = 0.9828) compared against IFS (AUC = 0.9959) to discriminate against MCI and controls, where in both cases, the AUC of the RUDAS-PE performed slightly less than the MMSE and IFS, respectively. Figure 4 shows the differential distribution of the RUDAS-PE according to the scores for each of the diagnostic groups.

#### Diagnostic Accuracy

The ability of the RUDAS-PE to discriminate between controls and patients with MCI (AUC: 0.98, 95%CI: 0.96–1.00) was slightly less than the IFS (0.99, 95% CI: 0.98–1.00), however there was no significant difference between both groups (*p* = 0.232). The ability of the RUDAS-PE to correctly discriminate between controls and patients with MCI (AUC: 0.98, 95% CI: 0.96–1.00) was statistically superior to the MMSE (0.85, 95% CI: 0.79–0.91), (*p* < 0.05). On the other hand, to discriminate between patients with MCI and dementia, the AUC of the IFS (1.00 95%CI: 0.99–1.00) and the MMSE (1.00, 95% CI: 0.99–1.00) were similar; both were slightly superior to the RUDAS-PE (0.98, 95% CI: 0.96–1.00), without significant difference (*p* = 0.312) (Table 3).

**Table 3:** Cut-off points and diagnostic performance for the Peruvian version of the Rowland Universal Dementia Assessment Scale (RUDAS-PE), INECO Frontal Screening (IFS) and Mini Mental State Examination (MMSE) to discriminate between controls and patients with mild cognitive impairment (MCI) and dementia.

**Diagnostic performance**	**Discrimination between controls and patients with MCI**			**Discrimination between patients with MCI and dementia**		
	**RUDAS-PE**	**IFS**	**MMSE**	**RUDAS-PE**	**IFS**	**MMSE**
Optimal cutoff point	23	22	19	19	18	14
Sensitivity, %	89.06	100	87.50	95.00	95.00	100
Specificity, %	93.33	93.3	65.00	96.83	96.83	98.41
Youden Index	0.82	0.93	0.53	0.92	0.92	0.98
Correctly classified, %	91.13	96.77	76.61	95.93	95.93	99.19
Likelihood ratio +	13.35	15.00	4.92	29.93	29.93	63.00
Likelihood ratio −	0.18	0.00	0.40	0.05	0.05	0.00
AUC (95% CI)	0.98 (0.96–1.00)	0.99 (0.98–1.00)	0.85 (0.79–0.91)	0.98 (0.96–1.00)	1.00 (0.99–1.00)	1.00 (0.99–1.00)

*Centros de Salud de Ventanilla, Callao, Lima. 2018-2019*.

*MCI, mild cognitive impairment; RUDAS-PE, Rowland Universal Dementia Assessment Scale, Peruvian version; IFS, INECO Frontal Screening; MMSE, Mini Mental State Examination; AUC, area under the curve; CI, confidence interval*.

The Youden Index was used to help derive optimal cutoffs to differentiate controls from MCI patients. A score of 23 was selected for the RUDAS-PE (sensitivity 89%, specificity 93%), and 22 for the IFS (sensitivity 100%, specificity of 93%), whereas 19 was the optimal cutoff for the MMSE with an acceptable sensitivity (87.5%) but high proportion of false positives (35%). At the same time, having a compatible MMSE for MCI, generates a small increase in the LR+ (4.92), and a minor decrease in LR- (0.40). Meanwhile in discriminating patients with MCI from dementia, the optimal cutoff was 19 for the RUDAS-PE (sensitivity 95%, specificity 96.83%); 18 for the IFS (sensitivity 95%, 96.83%); and 14 for the MMSE (sensitivity 100%, specificity 98.41%).

## Discussion

We have managed to validate the RUDAS from an entire sample of illiterate patients in an urban community; to date, there are only two RUDAS validation studies that include at least half of the illiterate population (13, 14, 21, 22). While another study conducted in Rio de Janeiro (23) included only 10% of illiterates in the AD group and 25.8% in the control group.

The internal consistency of the RUDAS-PE (Cronbach's alpha = 0.65) is in line with previous findings ranging from 0.54 to 0.80 (23, 24). The Spearman's correlation values highlight the usefulness of the RUDAS-PE as a significant predictor of cognitive and functional status confirming our initial findings in a population with a mid-level of education (15). We found a much higher value for the correlation between RUDAS-PE/MMSE (ρ = 0.86) with respect to those reported in previous literature indicating the RUDAS and MMSE correlation to fluctuate between 40 and 80% (25–30).

Based on the Youden index, the optimal cut-off point for the RUDAS-PE to discriminate patients with MCI and controls was 23, with better sensitivity (89%) and specificity (93%), percentage of correctly classified (91%) and LR+ (13) with LR- (0.18) as compared to the MMSE therefore supporting the diagnostic accuracy of the RUDAS-PE over the MMSE in discriminating controls from patients with MCI. These findings are similar to published studies comparing controls and patients with dementia (13–15, 21, 23, 25).

It is worth mentioning that the optimal cut-off scores for both the RUDAS-PE and MMSE in patients with illiteracy and low-levels of education vary from the scores found in literate populations with a mid-level of education (Table 4) (15). Taken together, these results would seem to suggest that the performance of the RUDAS-PE is influenced by level of education, but less so than the MMSE.

**Table 4:** Comparison of the RUDAS-PE cut-off scores for screening dementia in illiterate and literate populations.

	**Control vs. MCI**	**MCI vs. Dementia**
RUDAS-PE illiterate/low-education	23	19
RUDAS-PE literate/mid-education	24	21
MMSE illiterate/low-education	19	14
MMSE literate/mid-education	25	19

The probable explanations for the superiority of RUDAS-PE over MMSE in discriminating MCI and controls lie in the structure and weight given to the cognitive domains included in both BCTs. RUDAS-PE, unlike the MMSE, involves verbal fluency, visuospatial or body orientation, motor praxis, and judgment. Thus, assessing executive functions (verbal fluency, judgment) and motor praxis gives RUDAS-PE an advantage over the MMSE by being able to detect changes early in MCI (8–10); while alterations in orientation and attention/concentration occur in early or moderate stages of dementia (31, 32). In addition, administering the RUDAS-PE early could detect other types of dementia such as vascular dementia and the variants of fronto-temporal dementia that cannot be detected with the MMSE (33, 34). On the other hand, the weight attributed to the cognitive domains of the MMSE and RUDAS differs. Thus, while the MMSE concentrates its assessment on orientation, attention/concentration, and language the RUDAS gives greater weight to verbal, body orientation, and visuospatial praxis. This allows the RUDAS to detect different types of dementia syndrome (8, 15).

Limitations include sample size and implications for limited generalizability. A second limitation is the lack of longitudinal follow-up of each case in order to accurately establish the diagnoses of each patient group of our study. Thirdly, the diagnosis of dementia was based only on clinical judgment, without evidence of blood tests, brain images or biomarkers; pathological studies of brain samples could not be performed to establish a definitive diagnosis. A fourth limitation is that this study excluded patients from rural populations or populations whose predominant speech was other than Spanish.

In conclusion, the RUDAS-PE is an acceptable cognitive screening tool that has been validated in both illiterate/low-level of education and literate/ mid-level education populations. Our study proves its performance in discriminating controls from MCI to be superior to the MMSE and similar to both IFS and MMSE in discriminating MCI from dementia. Additionally, the RUDAS-PE is neither influenced by age or sex. Another advantage to the RUDAS-PE is its ease of administration, short application time, and minimal use of equipment. This screening tool has the potential to improve the diagnosis of MCI and dementia with diverse etiologies in the primary care setting.

## Recommendations

The link between lower educational achievement and socioeconomic disparities in LMICs is well-established. For developing countries like Peru whose demographic trends reflect a rapidly aging population it is imperative to identify and validate BCTs adapted for this group. An additional challenge lies in that the performance of illiterate individuals on neuropsychological tests often resembles that of literate individuals with dementia, which may contribute to misdiagnosis. We believe that our research will serve as a base for future studies on improving the quality of cognitive screening tools for dementia in low-educated settings. We recommend that further research should be undertaken in evaluating the RUDAS-PE in Peruvian populations that are not Spanish speaking, i.e., Quechua, Aymara, and other dialects. On a wider level, research is also needed to determine the performance of BCTs in primary care centers – where the rates of diagnostic errors tend to be highest, even in high income countries (35). We propose that further research should be undertaken in developing a differential diagnostic flowchart for geriatric populations focusing on cardiovascular and chronic disease risk factors for cognitive impairment including use of BCTs. In sum, our findings indicate the RUDAS-PE to be an appropriate tool for the discrimination of MCI and dementia in an illiterate and low-educated elderly population.

## Data Availability Statement

All datasets generated for this study are included in the article/Supplementary Material.

## Ethics Statement

The studies involving human participants were reviewed and approved by Instituto de Medicina Tropical “Daniel Alcides Carrión” of the “Universidad Nacional Mayor de San Marcos.” The patients/participants provided their written informed consent to participate in this study.

## Author Contributions

NC devised the project and main conceptual ideas. NC, RM, DL, and EH-P assisted in the study design. RM, KC, WR-G, JC, and CG collected the data. RM, EH-P, and WR-G organized the database. EH-P and TM performed the statistical analysis. NC, RM, and TM wrote the first draft of the manuscript. NC, RM, KC, MP-C, and TM wrote sections of the manuscript. All authors contributed to manuscript revision, read and approved the submitted version.

## Conflict of Interest

The authors declare that the research was conducted in the absence of any commercial or financial relationships that could be construed as a potential conflict of interest.
