# Supplementary material for: Validation of the RUDAS for the Identification of Dementia in Illiterate and Low-Educated Older Adults in Lima, Peru
Source: Front Neurol. 2020 May 5;11:374. doi: 10.3389/fneur.2020.00374 (PMC7232574; doi:10.3389/fneur.2020.00374)
Supplement: Supplementary file 1 [file Table_1.DOCX]

**Planilla de Juicio de Expertos**

Respetado juez: Usted ha sido seleccionado para evaluar el instrumento, prueba cognitiva breve, denominada Rowland Universal Dementia Assessment Scale (RUDAS) que forma parte de la investigación de tesis de doctorado en neurociencias “Validez del Rowland Universal Dementia Assessment Scale (RUDAS) para detectar deterioro cognitivo leve y demencia en estadios iniciales en población urbana iletrada de Lima”. La evaluación de los instrumentos es de gran relevancia para lograr que sean válidos y que los resultados obtenidos a partir de estos sean utilizados eficientemente; aportando tanto al área investigativa de la neurología cognitiva como a sus aplicaciones.

Agradecemos su valiosa colaboración.

NOMBRES Y APELLIDOS DEL JUEZ:

______________________________________________________________________

FORMACION ACADÉMICA

______________________________________________________________________

AREAS DE EXPERIENCIA PROFESIONAL ______________________________________________________________________

TIEMPO_______________________________________________________________

CARGO ACTUAL _______________________________________________________

______________________________________________________________________

INSTITUCION__________________________________________________________

Objetivo de la investigación:

Determinar la validez del RUDAS en la detección del deterioro cognitivo leve y la demencia en personas urbanas iletradas del Callao.

Objetivo del juicio de expertos:

Evaluar la adaptación cultural tras una adecuada conceptualización y operacionalización del constructo RUDAS.

Objetivo de la prueba RUDAS:

Intenta discriminar el nivel cognitivo para establecer individuos urbanos iletrados cognitivamente sanos, de pacientes con deterioro cognitivo leve y demencia.

De acuerdo con los siguientes indicadores califique cada uno de los ítems (memoria, orientación visuo-espacial, praxias, construcción visuo-espacial, juicio y lenguaje) según corresponda.

| CATEGORÍA | CALIFICACIÓN | INDICADOR |
| --- | --- | --- |
| SUFICIENCIA | 1. No cumple con el criterio  2. Bajo nivel  3. Moderado nivel  4. Alto nivel | Los ítems no son suficientes para medir la dimensión.  Los ítems miden algún aspecto de la dimensión pero no corresponden con la dimensión total.  Se deben incrementar algunos ítems para poder evaluar la dimensión completamente.  Los ítems son suficientes. |
| CLARIDAD | 1. No cumple con el criterio  2. Bajo nivel  3. Moderado nivel  4. Alto nivel | El ítem no es claro  El ítem requiere bastantes modificaciones o una modificación muy grande en el uso de las palabras de acuerdo con su significado.  Se requiere una modificación muy específica de algunos de los términos del ítem.  El ítem es claro, tiene semántica y sintaxis adecuada. |
| COHERENCIA | 1. No cumple con el criterio  2. Bajo nivel  3. Moderado nivel  4. Alto nivel | El ítem no tiene relación lógica con la dimensión.  El ítem tiene una relación tangencial con la dimensión.  El ítem tiene una relación moderada con la dimensión que está midiendo.  El ítem se encuentra completamente relacionado con la dimensión que está midiendo. |
| RELEVANCIA | 1. No cumple con el criterio  2. Bajo nivel  3. Moderado nivel  4. Alto nivel | El ítem puede ser eliminado sin que se vea afectada la medición de la dimensión.  El ítem tiene alguna relevancia, pero otro ítem puede estar incluyendo lo que mide éste.  El ítem es relativamente importante.  El ítem es muy relevante y debe ser incluido. |

Estimado Juez: coloque la calificación (1, 2, 3 o 4) en cada categoría, y sienta la total libertad para anotar las observaciones para cada dimensión del RUDAS

| **DIMENSIÓN** | **ÍTEM** | **SUFICIENCIA** | **COHERENCIA** | **RELEVANCIA** | **CLARIDAD** | **OBSERVACIONES** |
| --- | --- | --- | --- | --- | --- | --- |
| **Memoria (Registro)** | Café |  |  |  |  |  |
|  | Aceite |  |  |  |  |  |
|  | Huevos |  |  |  |  |  |
|  | Jabón |  |  |  |  |  |
| **Orientación visuo-espacial** | Enseñar o indicar partes del cuerpo |  |  |  |  |  |
| **Praxias motoras** | Alternancia de postura de manos |  |  |  |  |  |
| **Construcción visuo-espacial** | Copia del dibujo del cubo |  |  |  |  |  |
| **Juicio** | Situación hipotética de cruzar calle con mucho tráfico |  |  |  |  |  |
| **Memoria**  **(Recuerdo)** | Recordar la lista de cosas (café, aceite, huevos y jabón) |  |  |  |  |  |
| **Lenguaje** | Fluencia de categoría animal |  |  |  |  |  |

Estimado juez, considera que existe alguna dimensión que forma parte del constructo y que no está siendo evaluada? _______________________________________________

Cual?________________________________________________________________________________________________________________________________________

Fecha: Firma
