# Supplementary material for: Validation of the RUDAS for the Identification of Dementia in Illiterate and Low-Educated Older Adults in Lima, Peru
Source: Front Neurol. 2020 May 5;11:374. doi: 10.3389/fneur.2020.00374 (PMC7232574; doi:10.3389/fneur.2020.00374)
Supplement: Supplementary file 2 [file Table_2.DOCX]

**ROWLAND UNIVERSAL DEMENTIA ASSESSMENT SCALE (RUDAS)**

*Storey, Rowland, Basic, Conforti & Dickson. International Psycogeriatrics 2004;16(1):13-31*

**MEMORIA**

1. (REGISTRO) Quiero que imagine que vamos a una tienda. Tengo aquí la lista de la compra. Quiero que recuerde las cosas que necesitamos traer de la tienda. Cuando lleguemos a la tienda, dentro de 5 minutos más o menos, le preguntaré qué tenemos que comprar. Usted debe recordar esta lista: **CAFÉ, ACEITE, HUEVOS, JABÓN**. Por favor repita la lista. (PIDA A LA PERSONA QUE REPITA LA LISTA 3 VECES, SI LA PERSONA NO REPITE TODAS LAS PALABRAS, REPÍTALE LA LISTA HASTA QUE LAS HAYA APRENDIDO Y PUEDA REPETIRLAS, O, HASTA UN MÁXIMO DE 5 VECES).

**ORIENTACIÓN VISUO-ESPACIAL**

2. Le voy a pedir que me enseñe/indique diferentes partes del cuerpo. (Correcto=1). UNA VEZ LA PERSONA LOGRE 5 ACIERTOS EN ESTE APARTADO, NO CONTINÚE, YA QUE LA MÁXIMA PUNTUACIÓN ES 5.

(1) Enséñeme su pie derecho…………………………………………………………………….........1

(2) Enséñeme su mano izquierda…………………………………………………………….…………1

(3) Con su mano derecha toque su hombro izquierdo………………………………………..1

(4) Con su mano izquierda toque su oreja derecha…………………………………………….1

(5) Señale (indique cuál es) mi rodilla izquierda…………………………………………………1

(6) Señale (indique cuál es) mi codo derecho……………………………………………………..1

(7) Con su mano derecha señale (indique cuál es) mi ojo izquierdo……………………1

(8) Con su mano izquierda señale (indique cuál es) mi pie izquierdo………………….1

**……../5**

**PRAXIAS**

3. Le voy a mostrar un movimiento/ ejercicio/ juego con mis manos. Quiero que me mire e imite lo que yo haga. Imíteme haciendo esto. (UNA MANO CON EL PUÑO CERRADO, LA PALMA DE LA OTRA APOYADA SOBRE LA MESA, SE VAN ALTERNANDO LAS POSTURAS DE AMBAS MANOS). Ahora hágalo conmigo. Ahora quiero que usted siga haciendo este ejercicio a esta velocidad hasta que le diga que pare. (HACER LA DEMOSTRACIÓN A UN RITMO MODERADO POR 10 SEGUNDOS). EL EVALUADO DE REALIZAR EL EJERCICIO POR APROXIMADAMENTE 10 SEGUNDOS.

PUNTUAR COMO:

NORMAL **= 2** (muy pocos errores, en el caso que cometa alguno, ella misma los corrige, lo hace progresivamente mejor; mantiene bien la continuidad del movimiento; sólo muy ligeras pérdidas de sincronización entre las dos manos).

PARCIALMENTE ADECUADO **= 1** (errores notables con algún intento de corregirse; intenta mantener la continuidad de la acción; sincronización pobre).

FALLIDO **= 0** (incapaz de realizar la tarea; ausencia de persistencia; no lo intenta en absoluto).

**……../2**

**CONSTRUCCIÓN VISUO-ESPACIAL**

4 . Por favor, copie este dibujo, exactamente tal como lo ve (MOSTRAR UNA HOJA A4 CON EL DIBUJO DE UN CUBO EN PERSPECTIVA CON ARISTA LATERAL DE 12 CM Y ÁNGULO DE 45 GRADOS).

PUNTUAR SEGÚN:

1. Ha hecho la persona un dibujo basado en un cuadrado?.................................1
2. Aparecen todas las líneas internas en su dibujo?.............................................1
3. Aparecen todas las líneas externas en su dibujo?............................................1

**……../3**

**JUICIO**

5. Usted está parado en la vereda de una calle que tiene mucho tráfico. No hay crucero peatonal ni semáforos. Dígame que haría para cruzar al otro lado de la calle sin peligro. (SI LA PERSONA DA UNA RESPUESTA INCOMPLETA QUE NO INCLUYA LAS DOS PARTES DE LA RESPUESTA, INCÍTELE: “Podría hacer alguna otra cosa?”)

APUNTE EXACTAMENTE LO QUE EL PACIENTE DIGA Y RODEE CON UN CÍRCULO LAS PARTES DE LA RESPUESTA QUE FUERON INCITADAS.

PUNTUACIÓN:

Indicó la persona que miraría si pasaban coches? (si=2; si, incitada=1; no=0)

Propuso la persona alguna otra medida de seguridad? (si=2; si, incitada=1; no=0)

**……../4**

**MEMORIA (reciente)**

1. (RECUERDO) Acabamos de llegar a la tienda. Recuerda la lista de cosas que necesita comprar? (INCITACIÓN: SI LA PERSONA NO LOGRA RECORDAR NINGÚN ELEMENTO DE LA LISTA, DECIRLE: “El primero era café”). 2 puntos por cada ítem que diga la persona que no le fuese recordado, usando sólo café como incitación.

Café………..2

Aceite……..2

Huevos……2

Jabón………2

**……../8**

**LENGUAJE**

6. Le voy a dar 1 minuto para que me diga tantos nombres diferentes de animales como pueda. Veamos cuantos animales distintos es capaz de nombrar en un minuto. (REPITA LAS INSTRUCCIONES SI ES NECESARIO). La puntuación máxima para este apartado es 8. Si la persona nombra 8 animales diferentes en menos de un minuto no es necesario continuar.

**……../8**
